# Diterpenoid Alkaloids and One Lignan from the Roots of *Aconitum pendulum* Busch

**DOI:** 10.1007/s13659-019-00227-y

**Published:** 2019-11-14

**Authors:** Jun Wang, Xian-Hua Meng, Tian Chai, Jun-Li Yang, Yan-Ping Shi

**Affiliations:** CAS Key Laboratory of Chemistry of Northwestern Plant Resources and Key Laboratory for Natural Medicine of Gansu Province, Lanzhou Institute of Chemical Physics, Chinese Academy of Sciences, Lanzhou, 730000 People’s Republic of China

**Keywords:** *Aconitum pendulum*, Alkaloids, Lignan, Anti-AD activity

## Abstract

**Abstract:**

Diterpenoid alkaloids have neroprotective activity. Herein, three napelline-type diterpenoid alkaloids **1**–**3**, two aconitine-type diterpenoid alkaloids **4**–**5**, and one isoquinline-type alkaloid **6**, as well as one lignan glycoside **7**, have been isolated from the roots of *Aconitum pendulum* Busch. Compounds **1** and **7** were new compounds, and their chemical structures were determined on the basis of nuclear magnetic resonance (NMR) spectra and mass spectrometry analysis. A ThT assay revealed that compound **2** showed significant disaggregation potency on the A*β*_1−42_ aggregates.

**Graphical Abstract:**

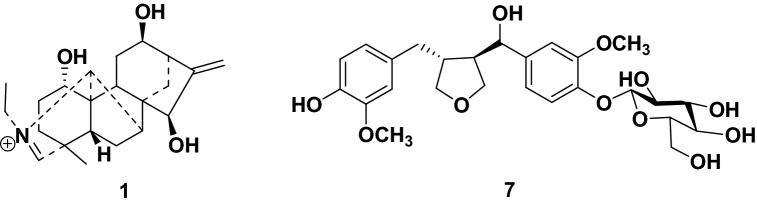

**Electronic supplementary material:**

The online version of this article (10.1007/s13659-019-00227-y) contains supplementary material, which is available to authorized users.

## Introduction

The plant *Aconitum pendulum* Busch, belonging to the Ranunculaceae family, is mainly distributed in Northwestern China at the altitude of 3000–4000 m [1]. The roots of *A. pendulum* have long been used as a traditional herb “Tie Bang Chui” for treating traumatic injury, fracture, rheumatism, and chilblains [2]. Previous studies showed that this plant mainly produced diterpenoid and norditerpenoid alkaloids [3], such as aconitine (AC), deoxyaconitine (DA), and mesaconitine (MA) [4], which were considered as the toxic components of this kind of folk medicine. These alkaloids have shown to cause widespread membrane excitation in cardiac, neural, and muscular tissues because of their significant activation on sodium channels [5–8]. Besides, they were also reported to show the potential as drug leads in Alzheimer diseases by targeting the neuronal nicotinic acetylcholine receptor [9–11].

**Fig. 1:**
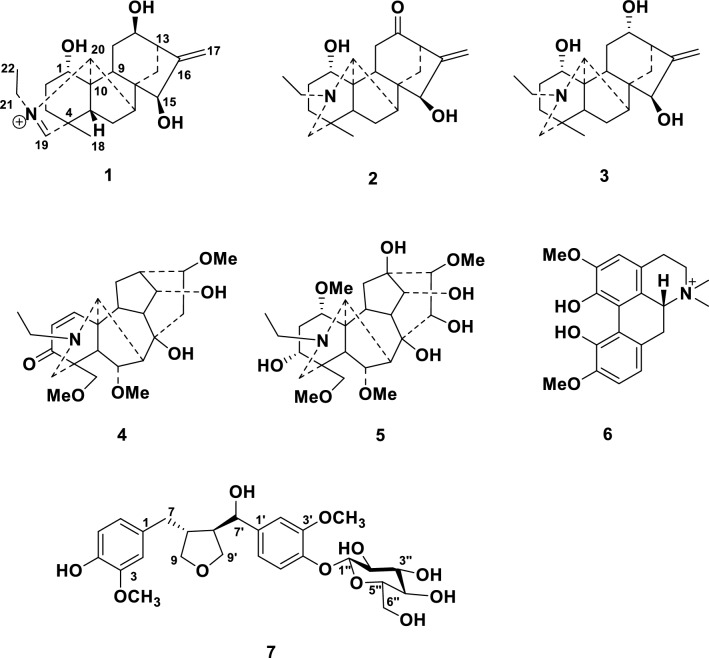
Molecular structures of compounds **1**−**7**

Our research group has long been focused on the discovery of bioactive natural compounds from the traditional herbs cultivated in Northwestern China [12–16]. As part of this ongoing program, a phytochemical study on the roots of *A. pendulum* collected from Gansu province has been conducted. Three napelline-type diterpenoid alkaloids **1**–**3**, two aconitine-type diterpenoid alkaloids **4**–**5**, and one isoquinline-type alkaloid **6**, as well as one lignan glycoside **7**, were isolated (Fig. 1). Herein, we report the isolation, structural determination, and biological activity of these isolates.

## Results and Discussion

Compound **1** was isolated as a colorless gum with a small value of specific optical rotatory value. Its chemical formula C_22_H_32_O_3_N was determined by the positive high-resolution MS at *m*/*z* 358.2362 (calcd. 358.2377). The IR spectrum indicated the existence of hydroxy (3396 cm^−1^) and olefinic (1678 cm^−1^) functionalities. The ^1^H and ^13^C NMR spectra of compound **1** demonstrated the presence of three oxygenated methine groups (*δ*_H_ 4.21, 4.14, 4.02; *δ*_C_ 77.2, 69.2, 68.8), one exocyclic double bond (*δ*_H_ 5.30, 5.16; *δ*_C_ 153.4, 113.3), a tertiary methyl group (*δ*_H_ 1.31; *δ*_C_ 21.3), an isolated iminium methine (*δ*_H_ 8.43; *δ*_C_ 184.8) [17], and three aliphatic quaternary carbons (*δ*_C_ 54.7, 52.3, 47.1). The data indicated that this compound was a C_20_-diterpenoid alkaloid possessing an iminium methine moiety [17]. Further HMBC and HSQC experiments determined that compound **1** shared the same molecular structure as that of aconicarmichinium A except for the relative configuration of OH-12 [17]. Specifically, three hydroxy groups were substituted at C-1, C-12, and C-15 based on the HMBC correlations from H-1 (*δ*_H_ 4.02) to C-2 (*δ*_C_ 31.9), C-3 (*δ*_C_ 35.5), C-5 (*δ*_C_ 45.0) and C-10 (*δ*_C_ 54.7), from H-12 (*δ*_H_ 4.14) to C-9 (*δ*_C_ 40.6), C-11 (*δ*_C_ 30.4), and C-13 (*δ*_C_ 44.9), from H-15 (*δ*_H_ 4.21) to C-7 (*δ*_C_ 52.2), C-8 (*δ*_C_ 52.3), C-9 (*δ*_C_ 40.6) and C-16 (*δ*_C_ 153.4). The position of Δ^16,17^ double bond was supported by the HMBC correlations from H_2_-17 (*δ*_H_ 5.30, 5.16) to C-13 (*δ*_C_ 44.9) and C-15 (*δ*_C_ 77.2). Moreover, the ^13^C NMR data of compound **1** and aconicarmichinium A [17] revealed that the major differences are the chemical shifts of C-12 (69.2 vs. 76.5), C-13 (44.9 vs. 48.6), C-14 (33.0 vs. 28.9), C-16 (153.4 vs. 158.5), and C-17 (113.3 vs. 109.6). This information suggested that the two compounds differed in the configuration of C-12, which was supported by the NOESY correlations from H-15 (*δ*_H_ 4.21) to H-14b (*δ*_H_ 1.28) and from H-14a (*δ*_H_ 1.78) to H-12 (*δ*_H_ 4.14). Therefore, the structure of **1** was named 12-*epi*-aconicarmichinium A chloride.

Compound **7** was purified as a white powder. The molecular formula C_26_H_34_O_11_ was determined by the positive high resolution MS at *m*/*z* 545.1992 ([M+Na]^+^, calcd. 545.1999). The IR spectrum suggested the presence of hydroxy (3360 cm^−1^) and benzene unit (1646, 1452, 1379 cm^−1^). Acid hydrolysis of **7** with 1 M HCl afforded d-glucose, based on gas chromatography analysis following treatment with l-cysteine methyl ester hydrochloride and trimethylsilylimidazole derivatization, and coupling pattern of the anomeric proton (d, *J* = 7.8 Hz) indicated a *β* configuration for the glucose unit. The ^1^H and ^13^C NMR data indicated the presence of a glucose unit (*δ*_H_ 4.86, 3.85, 3.67, 3.46, 3.46, 3.38, 3.38; *δ*_C_ 103.0, 78.4, 78.0, 75.1, 71.5, 62.7), two methoxyl groups (*δ*_H_ 3.85, 3.82; *δ*_C_ 56.9, 56.5), two tri-substituted phenolic rings (*δ*_H_ 7.12, 6.98, 6.87, 6.78, 6.70, 6.63; *δ*_C_ 151.0, 149.2, 147.4, 146.0, 139.7, 133.6, 122.3, 119.7, 118.1, 116.4, 113.5, 111.5), one oxygenated methine (*δ*_H_ 4.81; *δ*_C_ 84.0), and two oxygenated methylene (*δ*_H_ 3.99, 3.83, 3.72, 3.64; *δ*_C_ 73.8, 60.6). Detailed analysis of the NMR data indicated that compound **7** shared a similar structure with that of 3-(2,4-dihydroxy-3-methoxybenzyl)-4-(4-hydroxy-3-methoxybenzy1)tetrahydrofuran [18]. However, compound **7** had an additional sugar unit, which was substituted at C-4′ based on the HMBC correlation from H-1′' (*δ*_H_ 4.86) to C-4′ (*δ*_C_ 147.4). Due to the limited amount, the relative configuration at C-7′ remained undetermined. Therefore, compound **7** was determined as 3-(2,4-dihydroxy-3-methoxybenzyl-4-*O*-glucopyranosyl)-4-(4-hydroxy-3-methoxybenzy1)tetrahydrofuran.

On the basis of the literature data, the known compounds were determined as songorine (**2**) [19], napelline (**3**) [20, 21], ducloudine C (**4**) [22], aconine (**5**) [23], magnoflorine (**6**) [24].

Due to the available amounts, compounds **1**–**4** were evaluated for their anti-AD potential based on their effect on the copper-mediated A*β*_1−42_ disaggregation by using ThT assay [25] with resveratrol as positive control. All compounds were treated at 25 µM. As shown in Table 1, the disaggregate potency of compounds **1–4** were ranging from 10.2 ± 3.8 to 28.6 ± 2.9%, while the datum for the positive control resveratrol was 46.9 ± 4.6%. Compound **2** showed the most significant disaggregation effect on the A*β*_1−42_ aggregates.

**Table 1:** The disaggregation potency of compounds **1**–**4** (25 μM) on the Cu^2+^-induced A*β*_1−42_ aggregates

Compound	A*β*_1−42_ disaggregation (%)
**1**	10.2 ± 3.8
**2**	28.6 ± 2.9
**3**	17.4 ± 4.8
**4**	14.5 ± 1.5
Resveratrol	46.9 ± 4.6

## Experimental Section

### General

The optical rotation values were measured on a 241 polarimeter (Perkin-Elmer). The infrared spectra were measured by a FTS 165-IR instrument (Bio-Rad, USA). A Varian INOVA-400 FT-NMR spectrometer (USA) and a Bruker APEX II spectrometer were used to record the NMR and HRESIMS data, respectively. Different types of chromatographic materials were used for the fractionation of natural compounds, including Sephadex LH-20 (Amersham Biosciences), silica gel (200**–**300 mesh, Qingdao Haiyang Chemical Co., Ltd), and ODS (YMC Co., Ltd). Prep-HPLC separation was performed on a prep-HPLC manufactured by Hanbon Sci & Tech of China using a Megres C18 column (250 mm × 20 mm).

### Plant Materials

The roots of *A. pendulum* Busch (Ranunculaceae), collected from Gansu Province in China, were purchased from Lanzhou Huanghe Herbal Medicines Market in 2017. The materials were identified by Dr. Huan-Yang Qi at Lanzhou Institute of Chemical Physics (LICP), and a voucher specimen (ZY2017HTBC) was deposited at the CAS Key Laboratory of Chemistry of Northwestern Plant Resources.

### Extraction and Isolation

The air-dried roots (200 g) of *A. pendulum* were extracted with 90% ethanol/water (v/v) at room temperature (72 h × 3). After evaporated under reduced pressure, the residue (16.1 g) was suspended in water and successively partitioned with petroleum ether (PE), EtOAc, and *n*-BuOH (each 1.0 L × 3). The dried EtOAc part (3.9 g) was chromatographed over silica gel by eluting with gradient CH_2_Cl_2_/methanol (v/v, from 20:1 to 1:1) to yield 10 fractions (A1–A10) based on the TLC analysis. Fraction A3 (137 mg) was subjected to a Sephadex LH-20 column eluting with methanol to afford compounds **4** (16.3 mg) and **5** (2.2 mg). Fraction A5 (67 mg) was purified over ODS with methanol/water (v/v, from 50 to 90%) to afford compounds **1** (13.5 mg), **2** (10.0 mg), and **3** (15.1 mg). Fraction A7 (32 mg) was purified by HPLC eluting with methanol in water from 50 to 90% to yield compounds **6** (1.7 mg) and **7** (2.2 mg).

### 12-*epi*-Aconicarmichinium A chloride (1)

Colorless gum; $[\alpha ]_{D}^{20}$ − 2.3 (*c* 0.26, methanol); IR (neat) *v*_max_ 3396, 2923, 1678, 1200, 1132 cm^−1^; ^1^H (methanol-*d*_4_, 400 MHz) and ^13^C NMR (methanol-*d*_4_, 100 MHz) see Table 2; HRESIMS *m*/*z* 358.2362 [M]^+^ (calcd for C_22_H_32_NO_3_, 358.2377).

**Table 2:** NMR (400 MHz) spectroscopic data for Compound **1** in methanol-*d*_4_

Position	*δ*_H_ (*J* in Hz)	*δ*_C_	Position	*δ*_H_ (*J* in Hz)	*δ*_C_
1	4.02 overlap	68.8	12	4.14 overlap	69.2
2	1.97 m	31.9	13	2.79 dd (7.9, 4.3)	44.9
3	1.91 m 1.71 m	35.5	14	1.78 dd (13.3, 4.8) 1.28 m	33.0
4		47.1	15	4.21 br s	77.2
5	1.67 br d (7.1)	45.0	16		153.4
6	2.93 dd (13.8, 7.1) 1.36 m	26.4	17	5.30 s 5.16 s	113.3
7	2.46 d (3.8)	52.2	18	1.31 s	21.3
8		52.3	19	8.43 s	184.8
9	2.12 dd (11.7,7.1)	40.6	20	4.41 s	71.0
10		54.7	21	4.02 m	58.9
11	2.39 m 1.54 m	30.4	22	1.52 t (7.2)	14.2

### 3-(2,4-Dihydroxy-3-methoxybenzyl-4-*O*-glucopyranosyl)-4-(4-hydroxy-3-methoxybenzy1)tetrahydrofuran (7)

White powder; $[\alpha ]_{D}^{20}$ − 2.9 (*c* 0.37, methanol); IR (neat) *v*_max_ 3360, 2926, 1646, 1452, 1379, 1101, 1048 cm^−1^; ^1^H (methanol-*d*_4_, 400 MHz) and ^13^C NMR (methanol-*d*_4_, 100 MHz see Table 3; HRESIMS *m*/*z* 545.1992 [M+Na]^+^ (calcd for C_26_H_34_O_11_Na, 545.1993).

**Table 3:** NMR (400 MHz) spectroscopic data for Compound **7** in methanol-*d*_4_

Position	*δ*_H_ (*J* in Hz)	*δ*_C_	Position	*δ*_H_ (*J* in Hz)	*δ*_C_
1′		139.7	3-Me		56.5
2′	6.98 s (1H)	111.5	4		146.0
3′		151.0	5	6.70 d ( 8.0)	116.4
3′-OMe	3.85 s (3H)	56.9	6	6.63 d (8.0)	122.3
4′		147.4	7	2.51 dd (13.7, 11.4) 2.90 dd (13.7, 4.3)	33.8
5′	7.12 d (8.2)	118.1	8	2.70 m	44.0
6′	6.87 d (8.1)	119.7	9	3.99 m 3.72 m	73.8
7′	4.81	84.0	1″	4.86 d (7.8)	103.0
8′	2.34	54.3	2″	3.46 overlap	75.1
9′	3.83 m, 3.64 m	60.6	3″	3.46 t (8.8)	78.0
1		133.6	4″	3.38 overlap	71.5
2	6.78 s	113.5	5″	3.38 overlap	78.4
3	3.82 s	149.2	6″	3.85 overlap 3.67 overlap	62.7

### Acid Hydrolysis of Compound 7

This process was performed according to the literature [26]. The detailed process can be found in Supporting Information.

### ThT Assay

The procedures of ThT assay were reported in the literature [25]. The detailed information can be found in Supporting Information.

## Electronic supplementary material

Below is the link to the electronic supplementary material.

Supplementary material 1 (DOC 2403 kb)
