# Supplementary material for: Diterpenoid Alkaloids and One Lignan from the Roots of Aconitum pendulum Busch
Source: Nat Prod Bioprospect. 2019 Nov 14;9(6):419–23. doi: 10.1007/s13659-019-00227-y (PMC6872700; doi:10.1007/s13659-019-00227-y)
Supplement: Supplementary file 1 — Supplementary material 1 (DOC 2403 kb) [file 13659_2019_227_MOESM1_ESM.doc]

**Diterpenoid Alkaloids and One Lignan from the Roots of *Aconitum pendulum* Busch**

Jun Wang, Xian-Hua Meng, Tian Chai, Jun-Li Yang ^*^, and Yan-Ping Shi [[1]](#footnote-2)*

*CAS Key Laboratory of Chemistry of Northwestern Plant Resources and Key Laboratory for Natural Medicine of Gansu Province, Lanzhou Institute of Chemical Physics, Chinese Academy of Sciences, Lanzhou 730000, People’s Republic of China*

**For Compound 1**


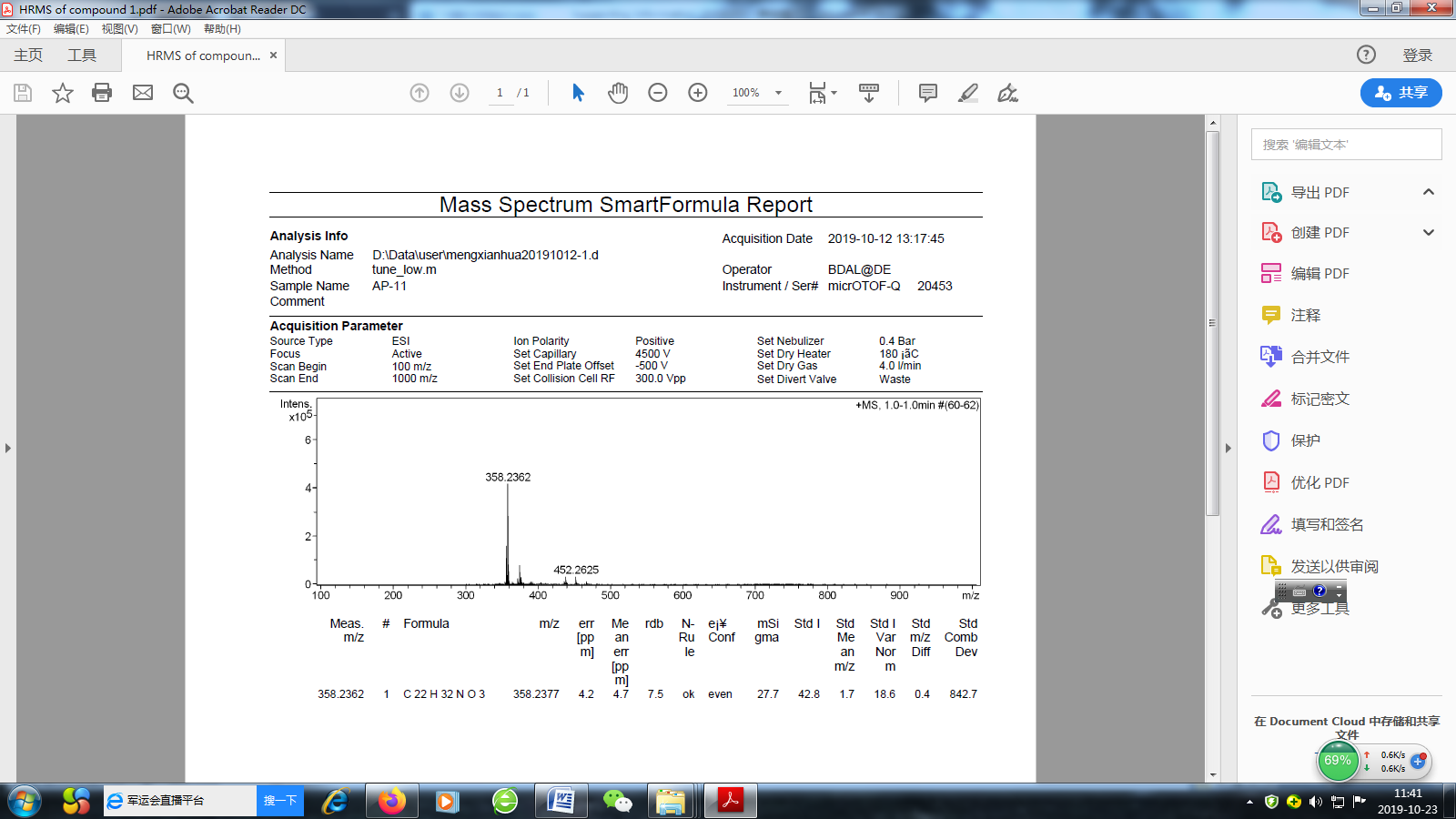


**Figure S1.** HRMS of compound **1**


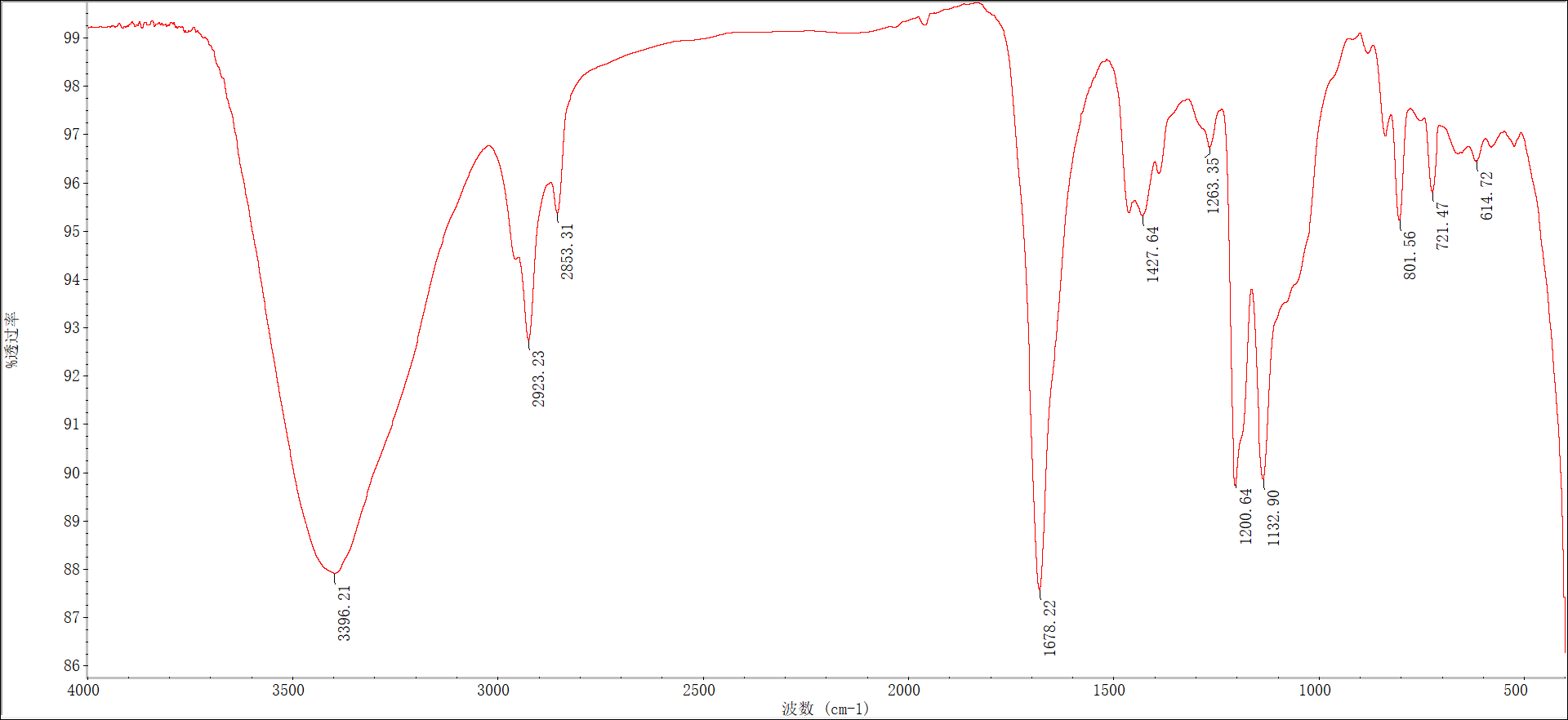


**Figure S2.** IR spectrum of compound **1**

**Figure S3.** ^1^H NMR Spectrum of **1** (400MHz, methanol-*d*_4_)

**Figure S4.** ^13^C NMR Spectrum of **1** (100MHz, methanol-*d*_4_)

**Figure S5.** HSQC Spectrum of **1** (400MHz, methanol-*d*_4_)

**Figure S6.** HMBC Spectrum of **1** (400MHz, methanol-*d*_4_)

**Figure S7.** NOESY Spectrum of **1** (400MHz, methanol-*d*_4_)

**For Compound 7**


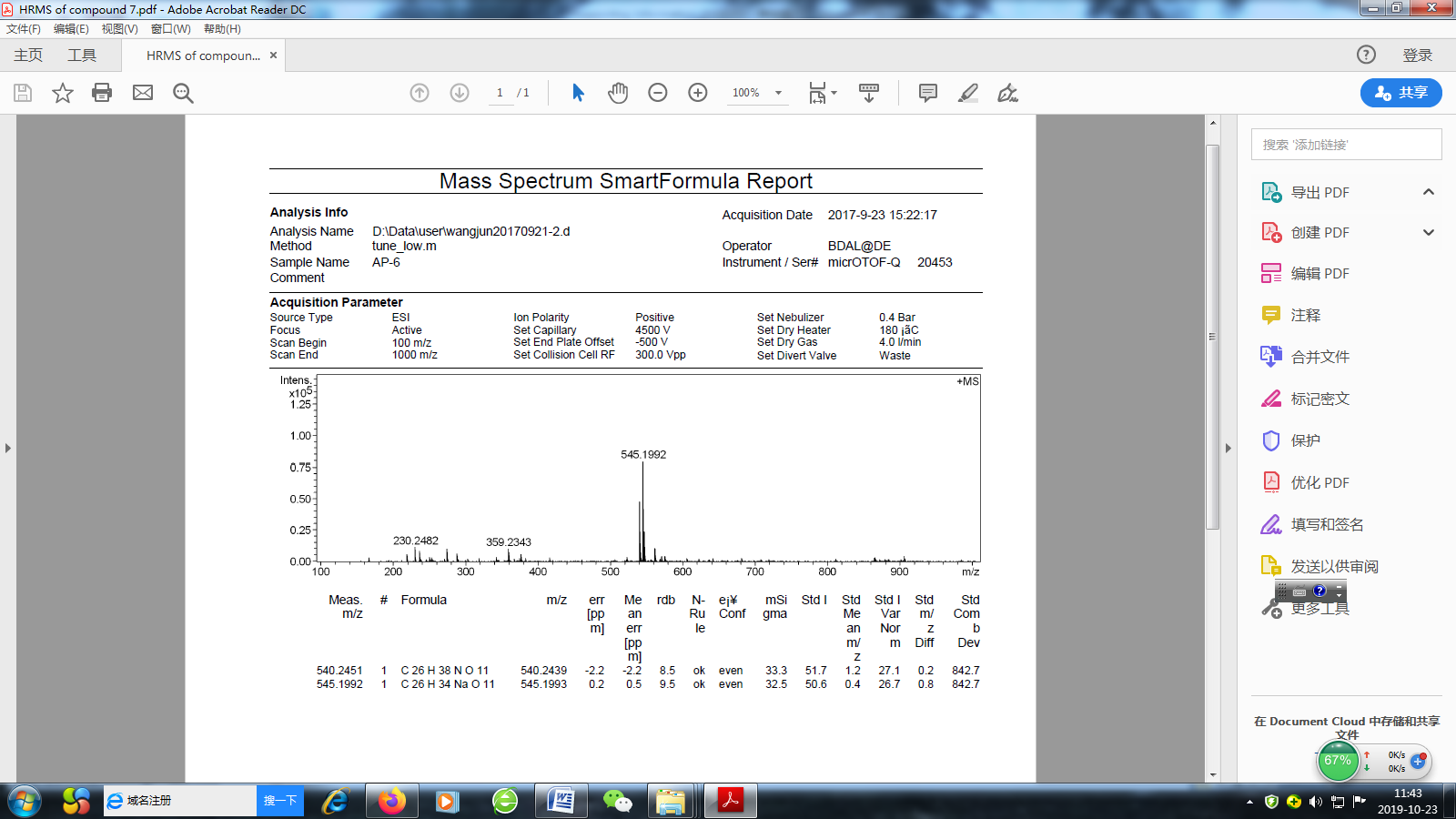


**Figure S8.** HRMS of compound **7**


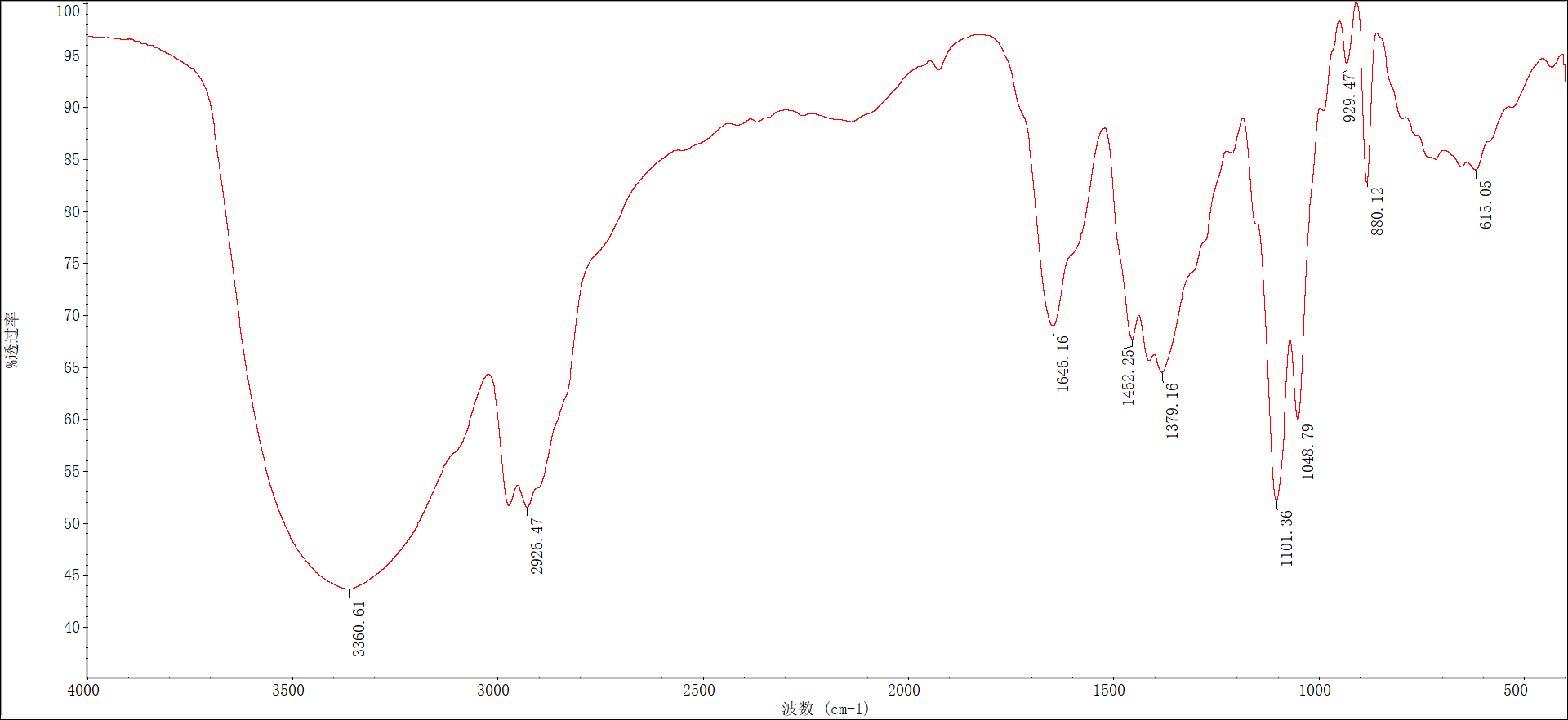


**Figure S9.** IR spectrum of compound **7**

**Figure S10.** ^1^H NMR Spectrum of **7** (400MHz, methanol-*d*_4_)

**Figure S11.** ^13^C NMR Spectrum of **7** (100MHz, methanol-*d*_4_)

**Figure S12.** HSQC Spectrum of **7** (100MHz, methanol-*d*_4_)

**Figure S13.** HMBC Spectrum of **7** (100MHz, methanol-*d*_4_)

**Acid Hydrolysis of Compound 7**. The new lignin glycoside **7** (1 mg) were hydrolyzed by refluxing with 1 M HCl (H_2_O/ethylene oxide, 1:1, 2 mL) for 3 h, followed by a partitioning between ethyl-acetate and water. The water residue was dissolved in pyridine (1 mL) and mixed with L-cysteine methyl ester hydrochloride (2 mg), and this mixture was kept at 60 ^o^C for 2 h and 0.2 mL of trimethylsilylimidazole was added thereafter, and again maintained at 60 ^o^C for another 2 h. Then, the residue was dried and partitioned between waterand *n*-hexane, and the *n*-hexane layer was analyzed by Gas Chromatographic system (detector: FID; detector temperature: 280 ^o^C; injection temperature, 250 °C; capillary column: DB-5, 30 m × 0.25 mm × 0.25 *μ*m; column temperature: 100 °C for 2 min and then increase to 280 °C at a rate of 10 °C/min; final temperature, 280 °C for 5 min; carrier gas: N_2_). In the same way, the trimethylsilyl-L-cysteine derivative of authentic sugar was prepared. By comparing the retention time (19.50 min) of the derivatives of compound **7** and authentic sugar, the absolute configurations of sugar components was determined as D-glucose.

**ThT Assay**

The dried A*β*_1−42_ peptide powder (purity ≥95%, Aladdin, Shanghai, China) was dissolved in 1,1,1,3,3,3-hexafluoro-2-propanol (HFIP) at a concentration of 1.0 mg∙mL^−1^. The HFIP/peptide solution was shaked at room temperature for 6 hours to gain the monomeric form of A*β*_1-42_. Then, the HFIP/peptide solution was dried under a gentle stream of nitrogen gas for 2 hours. The dried peptide power was then dissolved with anhydrous DMSO and stored at −20 °C. For the test of copper-mediated A*β*_1−42_ inhibition and disaggregation, the procedures were carried out according to the literature (J. Med. Chem. 2015, 58, 8616-8637). In brief, the above A*β*_1-42_ stock solution was diluted in 20 *μ*M HEPES (pH 6.6) with 150 *μ*M NaCl to the desired final concentration before use. Either 10 *μ*L of 25 *μ*M peptide solution and 10 *μ*L of test compound solution were incubated at 37 °C for 24 h together or the addition of peptide solution and compound solution was accomplished by two steps in a interval of 24 h, it was incubated at 37 °C for 24 h continuously. Then 180 *μ*L of 50 mM glycine-NaOH buffer (pH 8.0) containing thioflavin T (5 *μ*M) was added to dilute the above sample solution. After 5 min, the fluorescence intensities were measured (excitation, 450 nm; emission, 485 nm) by a fluorescence spectrophotometer (Horiba FluoroMax 4, USA). The percent inhibition of aggregation was calculated via the expression (1−IF_i_/IF_c_) × 100%, where IF_i_ and IF_c_ were the fluorescence intensities for A*β*_1-42_ in the presence and absence of test compounds after subtracting the background, respectively.

1. ^*^ Corresponding author, Tel.: +86-931-4968208; Fax: +86-931-4968094. *E-mail address*: [yangjl@licp.cas.cn](mailto:yangjl@licp.cas.cn) (J.-L. Yang), [shiyp@licp.cas.cn](mailto:shiyp@licp.cas.cn) (Y.-P. Shi) [↑](#footnote-ref-2)
